# Successful Intravascular Ultrasound-Guided Thrombus Removal and Angioplasty for Phlegmasia Cerulea Dolens in a Young Male With a History of Multiple Lower Extremity Deep Vein Thrombosis

**DOI:** 10.7759/cureus.39739

**Published:** 2023-05-30

**Authors:** Rutul Patel, Venkatachalam Mulukutla, Anuja Mahesh Mistry, Krupal Prajapati

**Affiliations:** 1 Internal Medicine, Texas Tech University Health Sciences Center El Paso, El Paso, USA; 2 Interventional Cardiology, The Hospitals of Providence, El Paso, USA; 3 Internal Medicine, Nathiba Hargovandas Lakhmichand (NHL) Municipal Medical College, Ahmedabad, IND

**Keywords:** angioplasty, intravascular ultrasound, venous stents, thrombus removal, deep venous thrombosis, phlegmasia cerulea dolens

## Abstract

Phlegmasia cerulea dolens is a rare and severe form of deep venous thrombosis (DVT), characterized by an extensive thrombus burden and compromised venous outflow. We present the case of a 28-year-old male with a history of bilateral lower extremity DVTs and multiple venous stents who presented with acute-onset pain and swelling in the left lower extremity. Diagnostic imaging confirmed an acute DVT extending throughout the left lower extremity, including the external iliac vein. Given the diagnosis of phlegmasia cerulea dolens, a multidisciplinary approach involving interventional cardiology, orthopedic surgery, and vascular surgery was adopted. Intravascular ultrasound (IVUS)-guided thrombus removal and angioplasty were performed to restore venous outflow and improve limb perfusion. The procedure successfully removed a significant amount of thrombus and improved flow throughout the venous system. The patient exhibited an excellent clinical response, with pain resolution and improved perfusion. This case highlights the challenges and effectiveness of a combined intervention in managing complex phlegmasia cerulea dolens cases with previous venous stents.

## Introduction

Phlegmasia cerulea dolens is a rare and severe form of deep venous thrombosis (DVT), characterized by an extensive thrombus burden and compromised venous outflow. It is associated with significant morbidity and mortality if not promptly recognized and managed. Cases with previous venous stents further complicate management due to under-expansion and the potential for thrombus formation within the stented segments [1].

Various interventions have been employed to address phlegmasia cerulea dolens, including systemic anticoagulation, catheter-directed thrombolysis, thrombus extraction, and venous stenting [2]. However, the optimal treatment strategy remains controversial, and outcomes can vary based on individual patient factors and the extent of the disease.

In this case report, we present a challenging case of phlegmasia cerulea dolens in a young male with a complex medical history of bilateral lower extremity deep vein thrombosis (DVT) and multiple venous stents. The patient presented with acute-onset pain, swelling, and signs of ischemia in the left lower extremity. Our management approach involved a multidisciplinary team, including interventional cardiology, orthopedic surgery, and vascular surgery, to address the extensive thrombus burden, under-expanded stents, and compromised venous outflow.

## Case presentation

**Figure 1:**
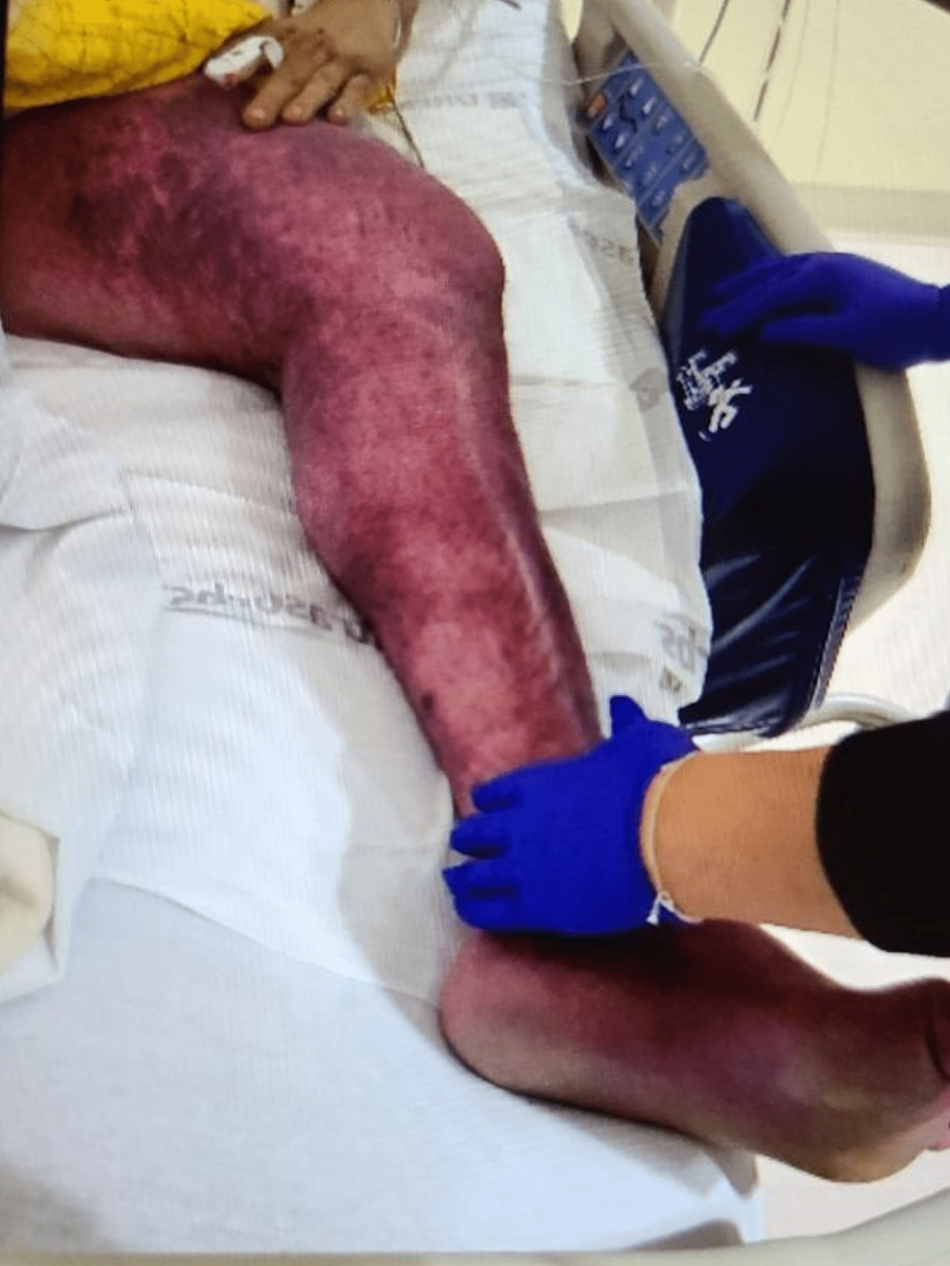
Patient's left lower extremity at presentation Physical examination of the left lower extremity demonstrating violaceous discoloration and edema.

A 28-year-old male with a history of bilateral lower extremity DVTs and multiple venous stents presented to the emergency department with acute-onset pain and swelling in the left lower extremity. The patient reported intense pain in the left lower limb, rated at 10/10 in intensity, along with significant edema and purple skin discoloration extending from the foot to the groin. On examination, the patient appeared to be in moderate distress due to pain. The left lower extremity exhibited significant edema, violaceous discoloration, and tenderness upon palpation (Figure 1).

The absence of left dorsalis pedis and posterior tibial pulses raised concerns about acute limb ischemia. However, Doppler ultrasound revealed good waveforms in the posterior tibial and dorsalis pedis arteries, suggesting preserved arterial flow. Left lower extremity venous duplex ultrasound confirmed the presence of acute deep venous thrombosis throughout the left lower extremity, extending to the external iliac vein, while the proximal extent was not visualized.

Given the diagnosis of phlegmasia cerulea dolens, the patient was admitted to the intensive care unit and initiated on an unfractionated heparin drip. Further imaging with CT angiography revealed enlargement and edema of the left thigh posterior and medial muscle compartments, possibly related to myositis or secondary to deep venous thrombosis. Additionally, asymmetric filling of the left lower leg arterial system compared to the contralateral right leg was noted, suggesting an inflammatory process.

Management and outcome

The patient's complex medical history, extensive thrombus burden, and under-expanded venous stents posed challenges in managing his condition. The diagnostic angiogram revealed no significant atherosclerosis or stenosis, but it did show very small lower extremity peripheral arteries with poor flow. The venogram revealed a severe thrombotic burden in the left iliac, femoral, and tibial arteries. Initial attempts at recanalization failed due to the heavy thrombus burden and under-expanded stents. Therefore, a multidisciplinary approach involving interventional cardiology, orthopedic surgery, and vascular surgery was adopted, and the patient underwent a fasciotomy to relieve compartment pressure and improve arterial flow.

Intravascular ultrasound-guided thrombus removal and angioplasty were performed to restore venous outflow and improve limb perfusion. During the procedure, we utilized intravascular ultrasound (IVUS) to assess the situation, revealing a substantial thrombus burden within the iliac and femoral venous stents, leading to compromised outflow. The stent at the entrance of the inferior vena cava (IVC) exhibited under-expansion, potentially causing the obstruction, along with telescoped and under-expanded segments. The IVUS also indicated a reduced luminal area on the left side where the stent was positioned, suggesting an associated outflow issue responsible for the obstruction. To address the situation, an 8-French sheath was introduced into the contralateral right popliteal vein, enabling IVUS evaluation using a 12-French sheath. To capture any existing clots, an INARI system disc (Inari Medical, Irvine, California) was deployed in the IVC.

**Figure 2:**
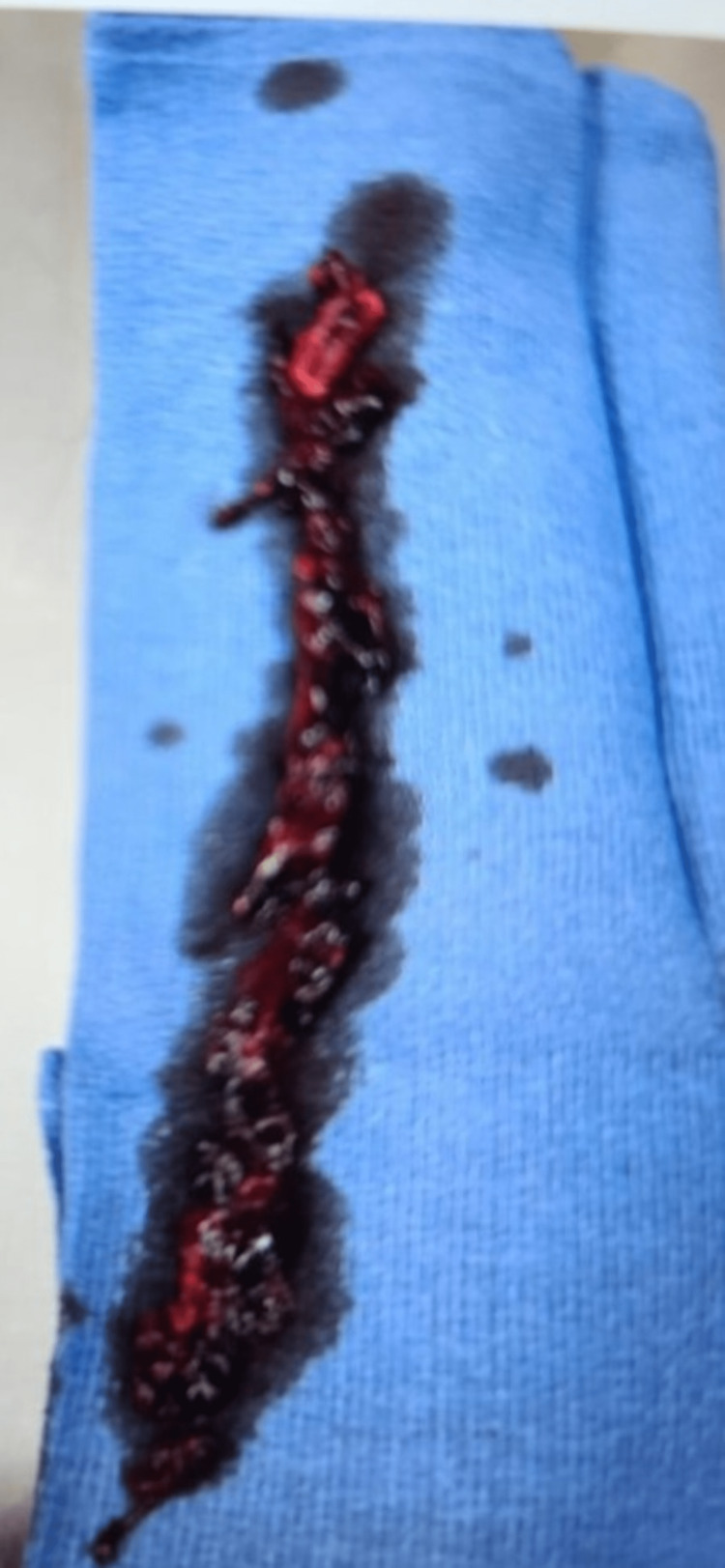
Demonstration of intra-procedural retrieved blood clots

We advanced to the left popliteal vein and deployed a 20-French flow retriever device. Through multiple passes across the popliteal, superficial femoral vein, femoral, iliac, and IVC, we successfully extracted a significant amount of thrombus and clot throughout the entire left leg using the INARI system (Figure 2).

**Figure 3:**
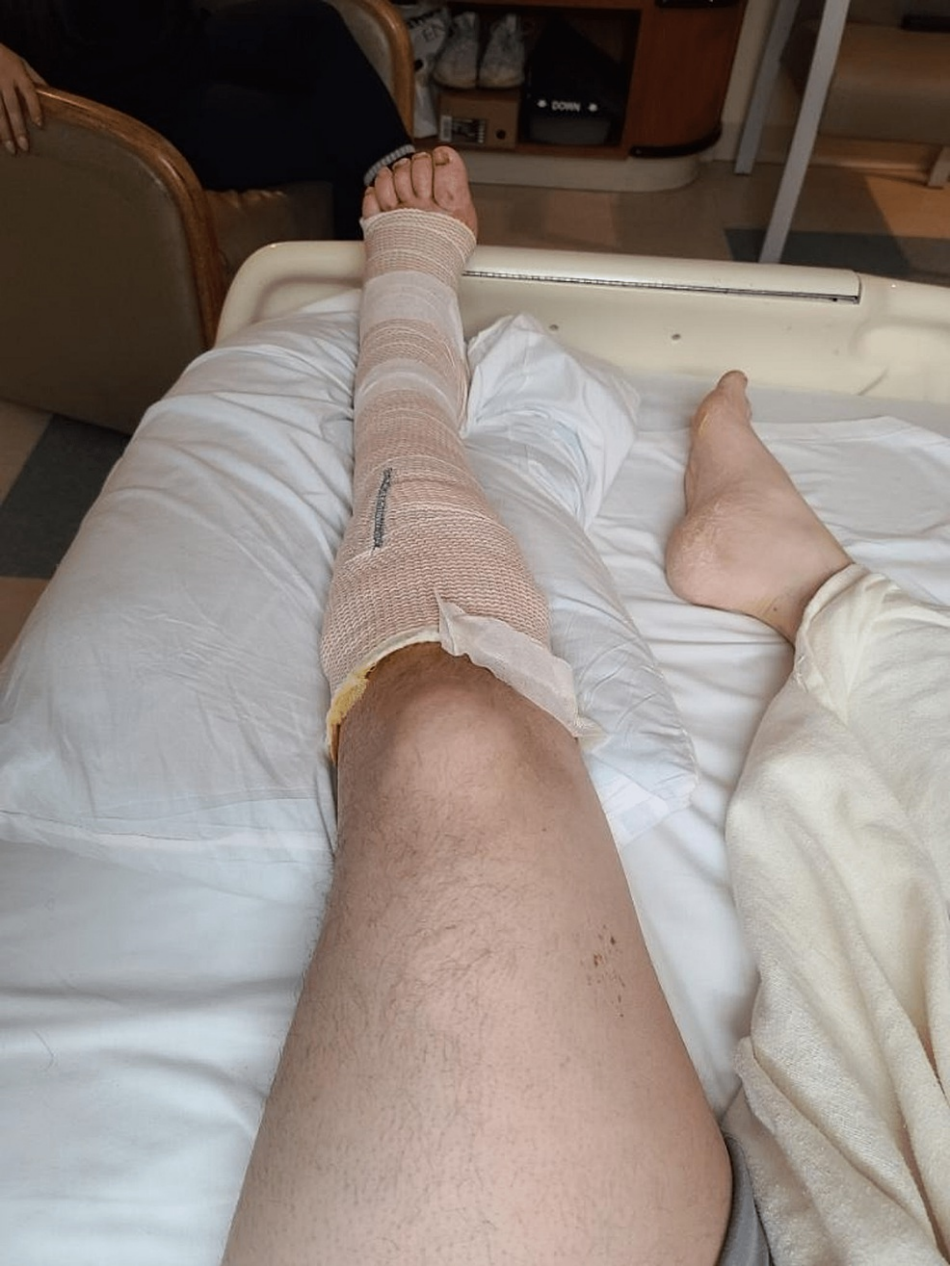
Left lower extremity examination a few days after the thrombectomy and angioplasty The physical examination reveals that the edema has resolved and the skin color of the left lower extremity has returned to normal.

Following these interventions, we observed restored outflow. To address the primary concern of poor outflow, we performed transluminal angioplasty using a 12 mm × 40 mm Atlas balloon along the entire stent. This procedure aimed to improve stent size and caliber, thereby enhancing outflow. After thrombus removal and angioplasty, the angiogram demonstrated a notable improvement in flow throughout the entire venous system. Post-intervention, the swelling and pain slowly resolved, and the posterior tibial and dorsalis pedis pulses were palpable. The violaceous discoloration also improved, and the patient showed an excellent clinical response (Figure 3). The patient was restarted on an unfractionated heparin drip, which was bridged to warfarin before discharge.

## Discussion

Phlegmasia cerulea dolens is a rare but critical condition that requires urgent intervention to restore venous outflow and prevent limb-threatening complications. The management of phlegmasia cerulea dolens has evolved over the years, and various interventions have been employed to address the complex pathophysiology and thrombus burden associated with this condition [3].

Systemic anticoagulation is the cornerstone of initial treatment for phlegmasia cerulea dolens. It aims to prevent thrombus propagation and reduce the risk of pulmonary embolism. However, anticoagulation alone may not be sufficient in cases with an extensive thrombus burden and compromised venous outflow [4]. In such cases, a multidisciplinary approach involving interventional cardiology, vascular surgery, and orthopedic surgery is crucial to restoring venous flow and salvaging the affected limb.

Catheter-directed thrombolysis has been used in the management of phlegmasia cerulea dolens to rapidly dissolve the thrombus and restore venous patency. It involves the infusion of thrombolytic agents directly into the affected vein under radiological guidance. However, this approach carries the risk of bleeding complications, especially in patients with recent surgery, active bleeding, or coagulopathy [5]. However, in our case, the extensive thrombus burden and under-expanded stents posed challenges that initially rendered catheter-directed thrombolysis unfeasible.

Thrombus removal using mechanical devices or aspiration techniques has emerged as an alternative or adjunct to thrombolysis in cases of phlegmasia cerulea dolens [6]. The use of IVUS-guided thrombus removal has shown promise in improving procedural success rates and reducing the risk of complications. IVUS allows real-time imaging of the vessel lumen, enabling accurate assessment of the thrombus burden and identification of under-expanded stents [7]. In our case, we performed IVUS-guided thrombus removal to address the extensive thrombus burden within the iliac and femoral venous stents, facilitating precise localization of the thrombus and enhancing the success of thrombus extraction.

Along with thrombus removal, angioplasty plays a crucial role in restoring venous outflow and optimizing limb perfusion in phlegmasia cerulea dolens. Transluminal angioplasty using balloons of appropriate size and length can effectively expand under-expanded stents and improve venous flow [8]. In our case, we employed a 12 mm × 40 mm Atlas balloon for transluminal angioplasty across the entire stent to enhance its size and caliber. This intervention led to a significant improvement in flow throughout the venous system, as evidenced by post-procedural angiography.

In cases of phlegmasia cerulea dolens, which is characterized by underlying venous stenosis or insufficient vascular diameter, venous stenting has been employed. Stents serve as structural support to prevent vessel collapse and maintain venous patency. However, in instances of extensive thrombus burden, the presence of under-expanded stents can further compromise venous outflow and contribute to the development of phlegmasia cerulea dolens [9]. In our case, we observed the under-expansion of venous stents at the entrance of the inferior vena cava, potentially leading to obstruction and thrombus formation. To address these issues and enhance stent functionality, we performed thrombus removal and angioplasty.

In our case, we utilized the INARI system, a mechanical thrombectomy device, to facilitate thrombus extraction. The INARI system comprises a retrievable nitinol disc designed to capture and remove thrombus from the venous system. It has demonstrated successful outcomes in various venous thromboembolic disorders, including phlegmasia cerulea dolens. By deploying the INARI system in the inferior vena cava, we achieved effective thrombus capture and extraction.

Phlegmasia cerulea dolens is a rare condition, and the available literature on its management is limited. However, various case reports and small studies have reported successful outcomes using different interventions, such as catheter-directed thrombolysis, thrombus extraction, angioplasty, and stenting [10]. The selection of the most appropriate intervention should be based on the patient's clinical presentation, thrombus burden, and the resources available.

## Conclusions

In conclusion, phlegmasia cerulea dolens is a rare and severe form of deep venous thrombosis that requires prompt recognition and a multidisciplinary approach for effective management. Our case highlights the challenges associated with previous venous stents and the successful utilization of intravascular ultrasound-guided thrombus removal, angioplasty, and the INARI system in restoring venous outflow and improving limb perfusion. Further research and larger studies are warranted to optimize treatment strategies and enhance outcomes in this uncommon and severe condition.

## References

[1] Cushman M (2007). Epidemiology and risk factors for venous thrombosis. Semin Hematol.

[2] Saha P, Humphries J, Modarai B (2011). Leukocytes and the natural history of deep vein thrombosis: current concepts and future directions. Arterioscler Thromb Vasc Biol.

[3] Vedantham S (2012). Interventional approaches to deep vein thrombosis. Am J Hematol.

[4] Kearon C, Akl EA, Ornelas J (2016). Antithrombotic therapy for VTE disease: chest guideline and expert panel report. Chest.

[5] Chang CT, Chang CD (2022). Successful treatment of phlegmasia cerulea dolens with percutaneous thrombectomy and catheter-directed thrombolysis: A case report. Medicine (Baltimore).

[6] Oguzkurt L, Ozkan U, Demirturk OS, Gur S (2011). Endovascular treatment of phlegmasia cerulea dolens with impending venous gangrene: manual aspiration thrombectomy as the first-line thrombus removal method. Cardiovasc Intervent Radiol.

[7] Vinha A, Pimenta J, Vasconcelos J, Maia M, Vidoedo J, Almeida Pinto J (2022). Percutaneous mechanical thrombectomy in phlegmasia cerulea dolens: case report and literature review. Port J Card Thorac Vasc Surg.

[8] O'Sullivan GJ, Semba CP, Bittner CA, Kee ST, Razavi MK, Sze DY, Dake MD (2000). Endovascular management of iliac vein compression (May-Thurner) syndrome. J Vasc Interv Radiol.

[9] Raju S, Tackett P Jr, Neglen P (2009). Reinterventions for nonocclusive iliofemoral venous stent malfunctions. J Vasc Surg.

[10] Vedantham S, Grassi CJ, Ferral H (2009). Reporting standards for endovascular treatment of lower extremity deep vein thrombosis. J Vasc Interv Radiol.

